# Draft Genome Sequence of *Geobacillus* sp. Strain FW23, Isolated from a Formation Water Sample

**DOI:** 10.1128/genomeA.00352-14

**Published:** 2014-05-08

**Authors:** Soham D. Pore, Preeti Arora, Prashant K. Dhakephalkar

**Affiliations:** Microbial Science Division, MACS’s Agharkar Research Institute, Pune, India

## Abstract

The thermophilic *Geobacillus* sp. strain FW23 was isolated from the Mehsana oil wells in Gujrat, India, during a screening for oil-degrading bacteria. Here, we report the draft genome sequence of *Geobacillus* sp. FW23, which may help reveal the genomic differences between this strain and the earlier reported species of the genus *Geobacillus*.

## GENOME ANNOUNCEMENT

High temperature and pressure make oil reservoirs an extreme environment that supports the growth of novel organisms which may possess unique biotechnological potential, such as production of thermostable enzymes. Investigation of diversity and functional analysis of microorganisms associated with oil reservoirs will provide assistance for the use of microorganisms as bioremediation agents in oil spills, which are a major source of environmental pollution (1).

FW23, a Gram-positive rod-shaped bacterial isolate with optimum growth at 60°C, was identified as a putative novel species of the genus *Geobacillus* based on 16S rRNA gene sequencing. The culture is deposited at the Microbial Culture Collection (MCC) (Pune, India) with the accession number MCC2339. Whole-genome sequencing was performed to enhance detailed understanding of the genome organization of this isolate and predict the presence of certain unprecedented genes involved in major pathways. Genomic DNA was isolated using a GenElute Bacterial DNA isolation kit (Sigma, USA). Whole-genome sequencing of *Geobacillus* sp. strain FW23 was performed using 316 chip and 200-bp chemistry on the Ion Torrent PGM platform (Life Technologies). Sequencing of the library generated 2,464,863 reads with an average read length of 228 bp. A total of 564 Mb of data were sequenced, with 485 Mb of data with a quality value above 20. Reads were assembled using MIRA assembler version 4.0.5. The coverage was 93.08×, and 227 contigs were generated. The length of the largest contig was 182,724 bp (*N*_50_, 39,806).

The genome was annotated using the RAST server (2), the KEGG database (3), GeneMark (4, 5), and Signal P4.1 (6). We predicted 3,844 coding sequences (CDS), 443 subsystems, 135 RNAs, and 204 signal peptides. A total of 127 unique genes associated with a subsystem in *Geobacillus* sp. FW23 were found when we performed a comparison with the nearest phylogenetic neighbor, *Geobacillus kaustophilus* HTA426.

Genes encoding several enzymes involved in oil degradation and utilization were detected using annotation studies. Metabolic pathways involved in the degradation of aromatic and aliphatic compounds such as toluene, xylene, naphthalene, dichlorobenzene, bromobenzene, dichloroethylene, and trichloroethylene were detected. In oil reservoirs, organic acids such as acetate have been found to be abundant and are commonly detected and recognized as important components for bacterial survival (7). In FW23, genes encoding acetate kinase, butyrate kinase, and formate dehydrogenase were found, and genes for the fatty acid metabolism pathway were also found. FW23 also has genes that encode methylmalonyl-coenzyme A (CoA) mutase. Ammonia, which is the primary nitrogen source in oil reservoirs (8), may be utilized by a specific ammonia transporter and assimilated via glutamate dehydrogenase or the glutamine synthetase pathway. Genes for sulfur metabolism proteins, such as alkanesulfonate monooxygenase, alkanesulfonate ABC transporter ATP-binding protein, and flavin mononucleotide (FMN) reductase, were present. Genes involved in copper tolerance and translocation, as well as arsenate reduction, were present. Genes involved in utilization of carbohydrate and amino acid synthesis, such as a gene for dihydropyriminidase, were detected. Information available from the whole-genome sequence enabled us to describe the metabolic profile of the strain and has revealed the metabolic potential of FW23 for oil degradation and the production of industrially important biomolecules such as thermostable enzymes.

### Nucleotide sequence accession numbers.

This whole-genome shotgun project has been deposited at DDBJ/EMBL/GenBank under the accession number JGCJ00000000. The version described in this paper is version JGCJ01000000.

## References

[1] Gogoi B Dutta N Goswami P Mohan T 2003 A case study of bioremediation of petroleum-hydrocarbon contaminated soil at a crude oil spill site. Adv. Environ. Res. 7:767–782. 10.1016/S1093-0191(02)00029-1

[2] Aziz RK Bartels D Best AA DeJongh M Disz T Edwards RA Formsma K Gerdes S Glass EM Kubal M Meyer F Olsen GJ Olson R Osterman AL Overbeek RA McNeil LK Paarmann D Paczian T Parrello B Pusch GD Reich C Stevens R Vassieva O Vonstein V Wilke A Zagnitko O 2008 The RAST server: rapid annotations using subsystems technology. BMC Genomics 9:75. 10.1186/1471-2164-9-75 18261238 PMC2265698

[3] Kanehisa M Goto S Kawashima S Okuno Y Hattori M 2004 The KEGG resource for deciphering the genome. Nucleic Acids Res. 32:277–280. 10.1093/nar/gkh063 PMC308797 14681412

[4] Besemer J Lomsadze A Borodovsky M 2001 GeneMarkS: a self-training method for prediction of gene starts in microbial genomes. Implications for finding sequence motifs in regulatory regions. Nucleic Acids Res. 29:2607–2618. 10.1093/nar/29.12.2607 11410670 PMC55746

[5] Lukashin AV Borodovsky M 1998 GeneMark.hmm: new solutions for gene finding. Nucleic Acids Res. 26:1107–1115. 10.1093/nar/26.4.1107 9461475 PMC147337

[6] Petersen TN Brunak S von Heijne G Nielsen H 2011 SignalP 4.0: discriminating signal peptides from transmembrane regions. Nat. Methods 8:785–786. 10.1038/nmeth.1701 21959131

[7] Barth T 1991 Organic acids and inorganic ions in waters from petroleum reservoirs, Norwegian continental shelf: a multivariate statistical analysis and comparison with American reservoir formation waters. Appl. Geochem. 6:1–15. 10.1016/0883-2927(91)90059-X

[8] Head IM Jones DM Larter SR 2003 Biological activity in the deep subsurface and the origin of heavy oil. Nature 426:344–352. 10.1038/nature02134 14628064

